# Temporal Trends in Anticoagulant Prescribing in Patients with Atrial Fibrillation According to Prior Ischemic Stroke or Transient Ischemic Attack: Insights from the CRAFT Registry

**DOI:** 10.3390/jcm15187242

**Published:** 2026-09-17

**Authors:** Janusz Bednarski, Michał Wojewódzki, Monika Szewczak, Marta Grzesiak, Maciej Karczewski, Karolina Własiuk, Emilia Kusy

**Affiliations:** 1 Clinic of Cardiology, Lazarski University, 02-662 Warsaw, Poland; 2 Department of Cardiology, St. John Paul II Western Hospital, 05-825 Grodzisk Mazowiecki, Poland

**Keywords:** atrial fibrillation, oral anticoagulation, direct oral anticoagulants, ischemic stroke, transient ischemic attack, prescribing patterns

## Abstract

**Introduction and Objectives**: Patients with atrial fibrillation (AF) and prior ischemic stroke or transient ischemic attack (TIA) are at high risk of recurrent thromboembolic events. Although direct oral anticoagulants (DOACs) have progressively replaced vitamin K antagonists (VKAs), it remains uncertain whether prior stroke/TIA influences anticoagulant prescribing or modifies temporal prescribing trends. **Methods**: This retrospective analysis included 3572 hospitalizations from the CRAFT registry between 2017 and April 2026. Hospitalization was the unit of analysis, and prior ischemic stroke/TIA status was determined at each admission. Anticoagulant prescribing was evaluated hierarchically as OAC versus no OAC, DOAC versus VKA, DOAC molecule selection, and, in exploratory analyses, within-molecule dose selection. Multivariable logistic and multinomial models included calendar year, age, sex, hypertension, diabetes mellitus, heart failure, AF type, and estimated glomerular filtration rate, with patient-level cluster-robust standard errors. Temporal trends and their interaction with prior stroke/TIA were assessed. Sensitivity analyses included categorical modeling of calendar year, exclusion of January–April 2026, and complete-case adjustment for atherosclerotic vascular disease. Benjamini–Hochberg FDR correction was applied to exploratory drug-specific comparisons. **Results**: Among 3572 hospitalizations, 437 (12.2%) involved patients with prior ischemic stroke/TIA. Prior stroke/TIA was not independently associated with OAC versus no OAC (OR 1.10, 95% CI 0.74–1.64; *p* = 0.643) or DOAC versus VKA selection (OR 0.68, 95% CI 0.45–1.01; *p* = 0.055). However, it was associated with overall DOAC molecule selection (global *p* = 0.0013), driven by a greater likelihood of dabigatran versus apixaban prescribing (RRR 1.87, 95% CI 1.29–2.72; *q* = 0.0052). In exploratory dose-specific analyses, prior stroke/TIA was associated with a lower likelihood of rivaroxaban 20 versus 15 mg prescribing (OR 0.54, 95% CI 0.33–0.90; *q* = 0.047). OAC prescribing increased over time (OR per year 1.16, 95% CI 1.09–1.23), as did DOAC versus VKA prescribing (OR per year 1.30, 95% CI 1.23–1.38; both *p* < 0.001), accompanied by a shift in DOAC molecule selection toward apixaban. Prior stroke/TIA did not significantly modify temporal prescribing trends, and sensitivity analyses supported the principal findings. **Conclusions**: Anticoagulant prescribing changed substantially between 2017 and April 2026, with increasing OAC and DOAC use and marked changes in DOAC molecule selection. Prior ischemic stroke/TIA was not independently associated with OAC use or DOAC versus VKA selection and did not significantly modify temporal prescribing trends, although differences were observed in DOAC molecule selection and exploratory dose-specific prescribing. Dose-specific findings describe prescribing patterns and should not be interpreted as assessments of dose appropriateness, efficacy, or safety.

## 1. Introduction

Atrial fibrillation (AF) is the most common sustained cardiac arrhythmia and an increasingly important public health challenge. The lifetime risk of developing AF is estimated at approximately one in three to one in five individuals aged ≥45 years, and its prevalence continues to rise with population aging and the growing burden of cardiovascular and non-cardiovascular comorbidities [1]. Ischemic stroke remains one of its most devastating complications.

More than 70,000 ischemic strokes occur annually in Poland and more than 1.1 million across Europe [2,3,4]. AF accounts for approximately 20–25% of ischemic strokes and therefore represents a major modifiable risk factor for cerebrovascular events [5,6].

AF-related stroke is associated with more severe neurological deficits, greater disability, and higher mortality than non-cardioembolic stroke [5,6,7,8]. This unfavorable prognosis reflects both the greater comorbidity burden of patients with AF and the pathophysiology of cardiac embolism, with larger emboli more likely to occlude major cerebral arteries and cause extensive cerebral infarction [5,6,7].

Oral anticoagulation is the cornerstone of stroke prevention in AF. Current European Society of Cardiology guidelines recommend direct oral anticoagulants (DOACs) for most patients requiring long-term anticoagulation and emphasize the timely initiation or resumption of anticoagulant therapy after ischemic stroke or transient ischemic attack (TIA) [8]. However, they do not recommend a specific oral anticoagulant solely on the basis of prior stroke/TIA. Over the past decade, anticoagulant prescribing has evolved substantially, driven by widespread DOAC adoption, updated guidelines, increasing clinical experience, and changes in reimbursement. Whether these changes have occurred similarly in patients with and without prior stroke/TIA, and whether a history of cerebrovascular events influences subsequent anticoagulant prescribing, remain uncertain.

We therefore evaluated temporal trends in anticoagulant prescribing according to prior ischemic stroke/TIA status in a contemporary real-world registry. We assessed whether prior stroke/TIA was independently associated with prescribing at successive levels of the anticoagulation pathway—including oral anticoagulation (OAC) use, DOAC versus vitamin K antagonist (VKA) selection, DOAC molecule selection, and, in exploratory analyses, within-molecule dose selection—and whether prior stroke/TIA modified these prescribing trends over time.

## 2. Methods

### 2.1. Study Design and Population

This retrospective observational study used fully anonymized data from the CRAFT registry (MultiCenter expeRience in AFib patients Treated with OAC; ClinicalTrials.gov identifier: NCT02987062), established to evaluate real-world anticoagulant prescribing in patients with AF. CRAFT was initiated jointly by the Department of Cardiology at the Medical University of Warsaw and St. John Paul II Western Hospital in Grodzisk Mazowiecki. Both centers enrolled patients between 2011 and 2016; since 2017, enrollment has continued exclusively at St. John Paul II Western Hospital, which contributed all observations included in the present analysis.

According to official guidance from the Bioethics Committee of the Medical University of Warsaw, retrospective studies using anonymized data do not require formal Ethics Committee approval; accordingly, no such approval was sought. Individual informed consent was not required because the analysis used fully anonymized, routinely collected clinical data. The study was conducted in accordance with the Declaration of Helsinki and relevant guidelines and regulations.

We included hospitalizations of patients with AF recorded between January 2017 and April 2026, regardless of whether OAC was prescribed at discharge. Registry recruitment was suspended in 2023, and data for 2026 cover January through April only. Patients receiving OAC for indications other than AF, including venous thromboembolism or mechanical prosthetic heart valves, were excluded. The registry includes demographic characteristics, cardiovascular risk factors, comorbidities, AF type, laboratory and echocardiographic data, and pharmacological treatment.

Prior ischemic stroke/TIA status was determined separately at each hospitalization based on the documented medical history. Only events occurring before the hospitalization being analyzed were classified as prior stroke/TIA; an acute ischemic stroke or TIA prompting or occurring during that hospitalization was not. Prior stroke/TIA status could therefore change between admissions if an event occurred in the intervening period.

Hospitalization was the unit of analysis because patient characteristics and treatment could change between admissions. Repeated hospitalizations were treated as separate observations, with patient-level cluster-robust standard errors used in all principal multivariable models to account for within-patient correlation.

The primary objective was to evaluate temporal trends in anticoagulant prescribing according to prior ischemic stroke/TIA status and to assess whether prior stroke/TIA was associated with prescribing or modified these trends over time.

### 2.2. Hierarchical Anticoagulant Prescribing Framework and Temporal Trend Analysis

Anticoagulant prescribing was evaluated hierarchically at four successive levels: (1) OAC versus no OAC in the overall population; (2) DOAC versus VKA among OAC-treated hospitalizations; (3) DOAC molecule selection, assessed using multinomial logistic regression with apixaban as the reference category for dabigatran and rivaroxaban; and (4) within-molecule dose selection: dabigatran 150 versus 110 mg, rivaroxaban 20 versus 15 mg, and apixaban 5 versus 2.5 mg. Rivaroxaban 10 mg prescriptions were excluded from the 20-versus-15-mg comparison. Edoxaban was not used in the study cohort and was therefore not included in the DOAC molecule analysis. Dose-specific analyses were exploratory and describe prescribing patterns rather than dose appropriateness or guideline concordance.

Annual prescribing proportions were calculated using analysis-specific denominators at each level of the hierarchy. Numerators, denominators, and 95% confidence intervals are reported in the Supplementary Materials.

Temporal trends were assessed in multivariable models with calendar year entered as a continuous variable to estimate the average annual change in prescribing. Interactions between calendar year and prior stroke/TIA were used to assess whether temporal trends differed according to cerebrovascular history. Sensitivity analyses treated calendar year as a categorical variable and repeated the continuous-year models after excluding January–April 2026.

### 2.3. Statistical Analysis

#### 2.3.1. Descriptive and Comparative Analyses

Descriptive analyses were performed using IBM SPSS Statistics 26.0 (IBM Corp., Armonk, NY, USA), while regression, cluster-robust variance, sensitivity, and false discovery rate analyses were performed using Python 3.13.5 with statsmodels 0.14.6. Categorical variables are presented as counts and percentages, and continuous variables as means ± standard deviations (SDs). Hospitalizations with and without prior stroke/TIA were compared using Pearson’s chi-square test for categorical variables and the Mann–Whitney U test for continuous variables. Descriptive comparisons used available-case data, with percentages calculated from observations with nonmissing values.

#### 2.3.2. Multivariable Hierarchical Models

The association between prior stroke/TIA and anticoagulant prescribing was assessed using binary logistic regression for OAC versus no OAC, DOAC versus VKA, and within-molecule dose comparisons, and multinomial logistic regression for DOAC molecule selection. Models included calendar year, age, sex, hypertension, diabetes mellitus, heart failure, AF type, and eGFR. Prior stroke/TIA was the exposure of interest in association models and a covariate in temporal-trend models; interaction models additionally included a calendar year × prior stroke/TIA term. The CHA_2_DS_2_-VA score was not included because prior stroke/TIA is a component of the score.

Patient-level cluster-robust standard errors were used in all principal association, temporal-trend, and interaction models to account for within-patient correlation due to repeated hospitalizations, while retaining hospitalization as the unit of analysis.

Atherosclerotic vascular disease was excluded from the principal models because its availability varied systematically across calendar years, and complete-case analysis would have disproportionately excluded recent observations. History of bleeding, anemia, and thrombocytopenia was also excluded because these data were not consistently available throughout the study period. No imputation was performed for these structurally unavailable variables. A complete-case sensitivity analysis additionally included atherosclerotic vascular disease in observations for which these data were available.

DOAC dose appropriateness was not assessed because Cockcroft–Gault creatinine clearance and all drug-specific dose-reduction criteria could not be consistently reconstructed. Within-molecule dose analyses therefore describe observed prescribing patterns only and should not be interpreted as assessments of appropriate or inappropriate dosing.

#### 2.3.3. Multiplicity and Reporting

The two molecule-specific contrasts and three within-molecule dose comparisons constituted a single exploratory family of five drug-specific comparisons. Benjamini–Hochberg false discovery rate (FDR) correction was applied to these comparisons and to the corresponding five calendar year × prior stroke/TIA interaction contrasts; nominal *p* values and FDR-adjusted *q* values are reported. Global DOAC molecule-selection tests and the principal hierarchical comparisons were not included in the FDR family.

Results from binary logistic regression are reported as odds ratios (ORs) and those from multinomial logistic regression as relative risk ratios (RRRs), with 95% confidence intervals (CIs). Global effects from multinomial models are reported using Wald chi-square statistics. All tests were two-sided. A nominal *p* value < 0.05 was considered statistically significant for principal comparisons, whereas exploratory drug-specific findings were interpreted using FDR-adjusted *q* values.

#### 2.3.4. Use of Generative Artificial Intelligence

During manuscript preparation and revision, the authors used ChatGPT (OpenAI) to assist with language editing and to improve clarity and consistency. Generative AI was not used to generate, modify, or interpret the primary study data or to make independent methodological or statistical decisions. All analyses, outputs, interpretations, and manuscript revisions were critically reviewed and verified by the authors, who take full responsibility for the content of the manuscript.

## 3. Results

### 3.1. Baseline Characteristics

Among 3572 hospitalizations involving 2781 unique patients, 437 (12.2%) involved patients with prior ischemic stroke/TIA. Of the 2781 patients, 569 (20.5%) had more than one hospitalization during the study period.

Patients with prior stroke/TIA were older and had a greater burden of cardiovascular comorbidities, including hypertension, diabetes mellitus, heart failure, coronary artery disease, previous myocardial infarction, and atherosclerotic vascular disease. They also had higher CHA_2_DS_2_-VA scores and lower mean eGFR. Detailed baseline characteristics are presented in Table 1.

### 3.2. Overall Anticoagulant Treatment Patterns

In unadjusted analyses, VKA (11.4% vs. 7.4%; *p* = 0.004) and dabigatran 110 mg use (10.5% vs. 6.8%; *p* = 0.007) were more frequent in hospitalizations involving patients with prior stroke/TIA, whereas rivaroxaban 20 mg use was less frequent (12.4% vs. 22.2%; *p* < 0.001). No other significant differences in anticoagulant prescribing were observed between groups. Detailed unadjusted prescribing patterns are presented in Table 2.

### 3.3. Hierarchical Association Between Prior Stroke/TIA and Anticoagulant Prescribing

In multivariable analyses accounting for patient-level clustering, prior stroke/TIA was not independently associated with OAC versus no OAC (adjusted OR 1.10, 95% CI 0.74–1.64; *p* = 0.643) or DOAC versus VKA selection (adjusted OR 0.68, 95% CI 0.45–1.01; *p* = 0.055). However, prior stroke/TIA was associated with overall DOAC molecule selection (global Wald χ^2^ = 13.22, df = 2; *p* = 0.0013), driven by a greater likelihood of dabigatran versus apixaban prescribing (RRR 1.870, 95% CI 1.286–2.718; *p* = 0.0010; FDR-adjusted *q* = 0.0052). Rivaroxaban versus apixaban prescribing did not differ significantly.

In exploratory within-molecule dose analyses, prior stroke/TIA was associated with a lower likelihood of rivaroxaban 20 mg versus 15 mg prescribing (OR 0.542, 95% CI 0.326–0.904; *p* = 0.0189; *q* = 0.0471), whereas no significant associations were observed for dabigatran or apixaban dose selection. Detailed results are presented in Table 3.

### 3.4. Temporal Changes in Anticoagulant Prescribing

Anticoagulant prescribing changed substantially over time. The odds of OAC prescribing increased by approximately 16% per year (OR 1.160, 95% CI 1.095–1.229; *p* < 0.001), and, among OAC-treated hospitalizations, the odds of DOAC versus VKA prescribing increased by approximately 30% per year (OR 1.303, 95% CI 1.232–1.378; *p* < 0.001). DOAC molecule selection also changed significantly (global Wald χ^2^ = 131.61, df = 2; *p* < 0.001), with decreasing relative likelihoods of both dabigatran versus apixaban (RRR 0.772, 95% CI 0.737–0.808; *q* < 0.001) and rivaroxaban versus apixaban prescribing (RRR 0.865, 95% CI 0.833–0.898; *q* < 0.001), indicating a shift toward apixaban.

In exploratory within-molecule dose analyses, the odds of rivaroxaban 20 versus 15 mg (OR 1.147, 95% CI 1.077–1.221; *q* < 0.001) and apixaban 5 versus 2.5 mg prescribing (OR 1.396, 95% CI 1.298–1.502; *q* < 0.001) increased over time, whereas no significant linear trend was observed for dabigatran 150 versus 110 mg (OR 1.062, 95% CI 0.974–1.159; *q* = 0.174).

**Figure 1:**
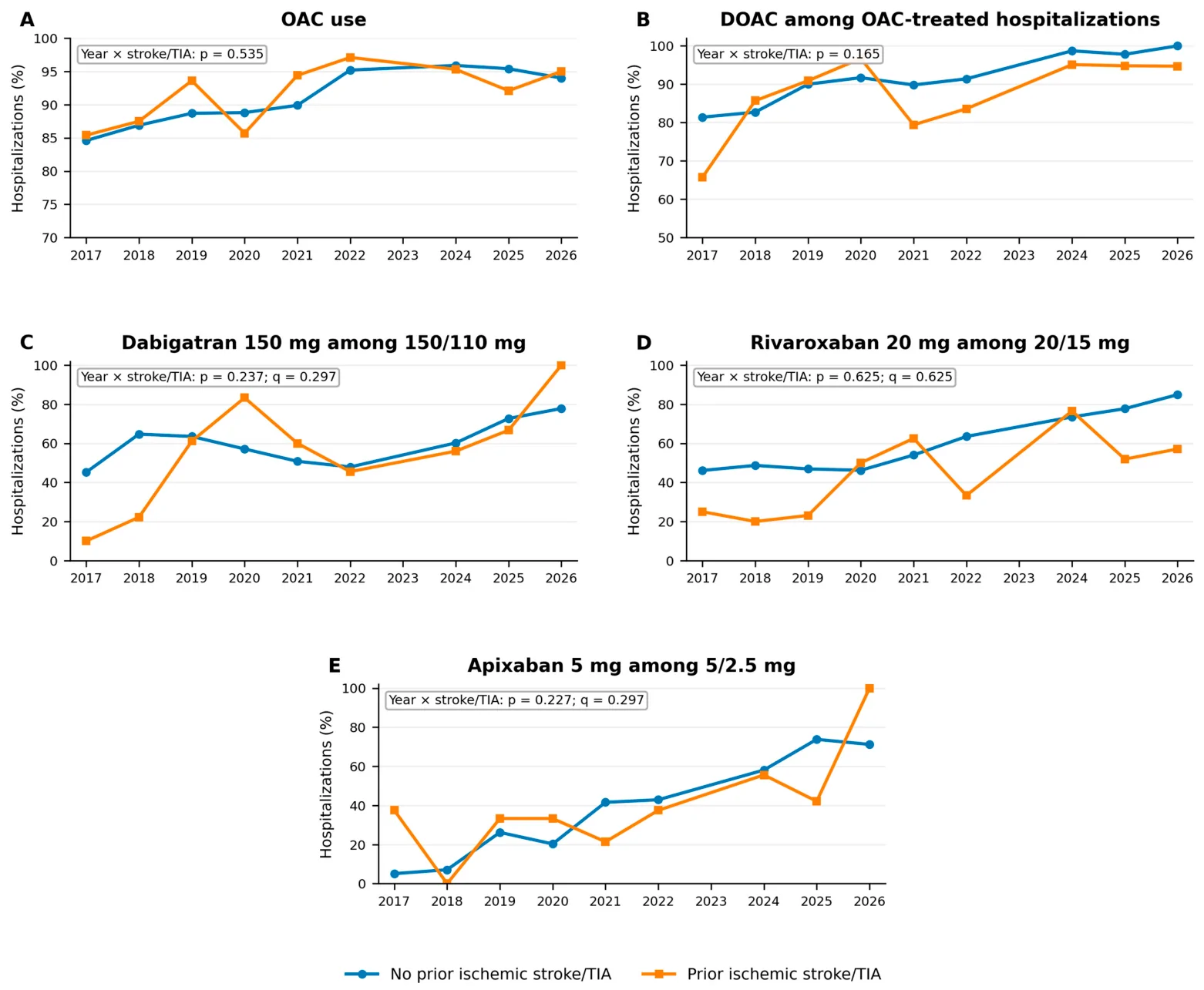
Annual anticoagulant prescribing patterns by prior ischemic stroke/TIA status. Annual observed prescribing proportions according to prior ischemic stroke/TIA status. Panel (**A**) shows OAC use among all eligible hospitalizations; Panel (**B**), DOAC use among OAC-treated hospitalizations; Panel (**C**), dabigatran 150 mg among hospitalizations treated with dabigatran 150 or 110 mg; Panel (**D**), rivaroxaban 20 mg among those treated with rivaroxaban 20 or 15 mg; and Panel (**E**), apixaban 5 mg among those treated with apixaban 5 or 2.5 mg. Interaction statistics were derived from multivariable models including calendar year × prior stroke/TIA, with patient-level cluster-robust standard errors. For exploratory dose comparisons (Panels **C**–**E**), nominal *p* values and FDR-adjusted *q* values are shown. None of the exploratory interaction contrasts remained significant after FDR correction. No observations were available for 2023 because registry recruitment was suspended; 2026 data cover January–April only. Rivaroxaban 10 mg was excluded from the 20-versus-15-mg comparison. Abbreviations: DOAC, direct oral anticoagulant; FDR, false discovery rate; OAC, oral anticoagulant; TIA, transient ischemic attack.

**Figure 2:**
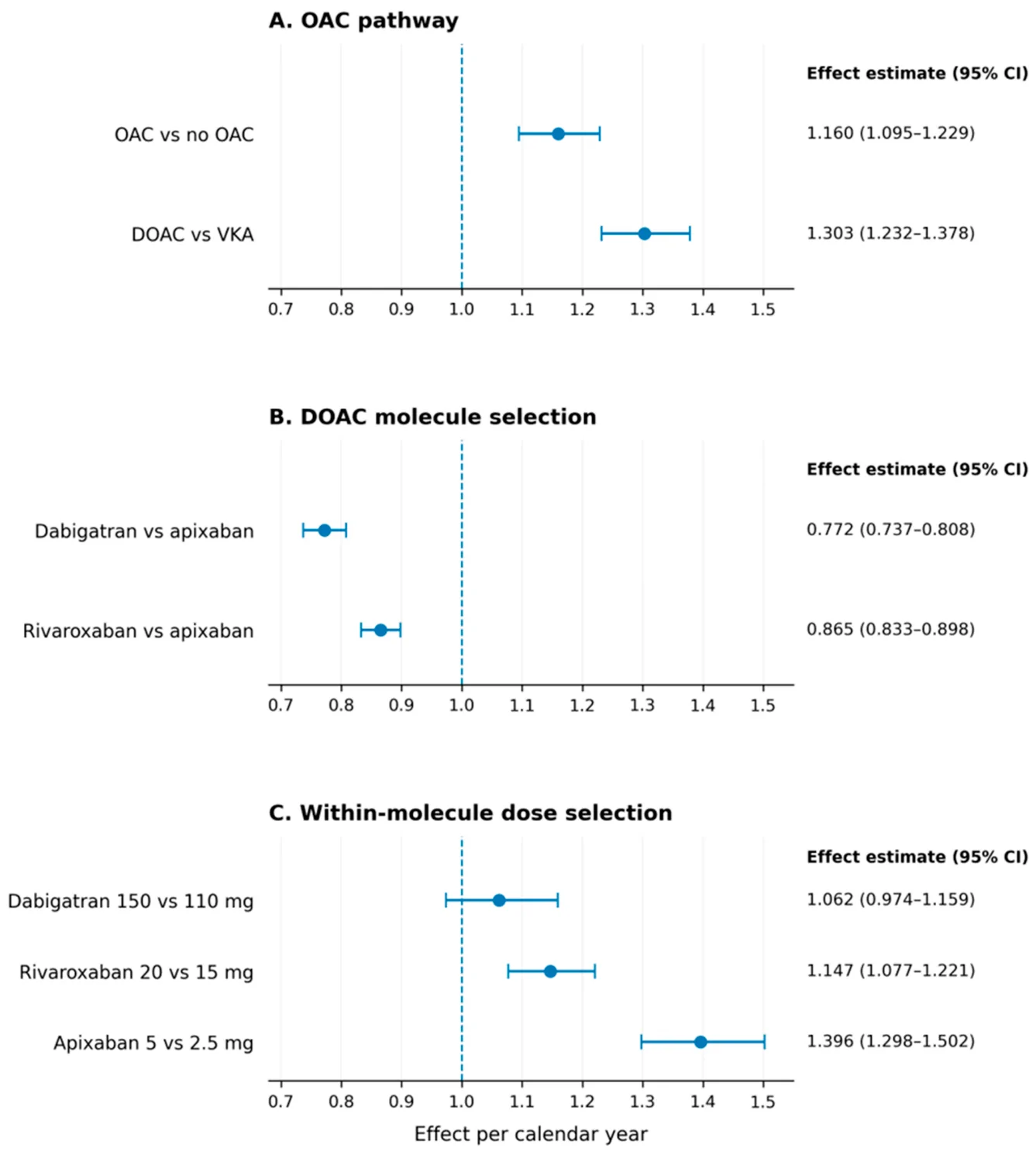
Adjusted temporal trends in hierarchical anticoagulant prescribing. Forest plot of adjusted temporal trends across successive levels of anticoagulant prescribing. Panel (**A**) shows OAC versus no OAC and DOAC versus VKA; Panel (**B**), DOAC molecule selection, with apixaban as the reference; and Panel (**C**), exploratory within-molecule dose comparisons. Effect estimates represent the change per one-year increase in calendar year and are reported as ORs or RRRs with 95% CIs. Models were adjusted for age, sex, hypertension, diabetes mellitus, heart failure, AF type, eGFR, and prior stroke/TIA, with patient-level cluster-robust standard errors. Benjamini–Hochberg FDR correction was applied to the five exploratory drug-specific comparisons. Rivaroxaban 10 mg was excluded from the 20-versus-15-mg comparison. The vertical dashed line indicates no temporal change (effect estimate = 1.0). Abbreviations: AF, atrial fibrillation; CI, confidence interval; DOAC, direct oral anticoagulant; eGFR, estimated glomerular filtration rate; FDR, false discovery rate; OAC, oral anticoagulant; OR, odds ratio; RRR, relative risk ratio; TIA, transient ischemic attack; VKA, vitamin K antagonist.

Adjusted temporal-trend estimates are presented in Table 4, annual observed proportions with analysis-specific denominators and 95% CIs in Supplementary Table S3, and the corresponding prescribing trajectories and adjusted effect estimates in Figure 1 and Figure 2, respectively.

### 3.5. Interaction and Sensitivity Analyses

There was no evidence that prior stroke/TIA modified temporal prescribing trends at any level of the hierarchical pathway. Interactions were not significant for OAC versus no OAC (*p* = 0.535), DOAC versus VKA (*p* = 0.165), or overall DOAC molecule selection (global *p* = 0.197), and none of the five exploratory molecule- or dose-specific interaction contrasts remained significant after FDR correction (Supplementary Table S1).

The principal temporal findings were robust when calendar year was treated as a categorical variable and when the partial January–April 2026 period was excluded. Temporal changes in OAC use, DOAC versus VKA selection, and DOAC molecule selection remained significant in both sensitivity analyses. Trends in rivaroxaban and apixaban dose selection also remained significant, whereas no significant linear trend was observed for dabigatran dose selection. However, the categorical-year analysis showed significant between-year heterogeneity, consistent with a non-linear pattern (Supplementary Table S2).

In an additional complete-case sensitivity analysis including atherosclerotic vascular disease, the direction and statistical interpretation of the principal association, temporal-trend, and interaction findings were materially unchanged (Supplementary Table S4).

## 4. Discussion

The present study provides a contemporary view of how anticoagulant prescribing in patients with AF has evolved during the mature DOAC era and whether these changes differ according to prior ischemic stroke/TIA. The major shifts in anticoagulant practice occurred largely irrespective of cerebrovascular history. Prior stroke/TIA was not associated with OAC use or DOAC versus VKA selection and did not significantly modify temporal prescribing trends. Differences emerged only at the level of individual DOAC selection and, in exploratory analyses, within-molecule dose selection. These findings suggest that prior cerebrovascular events have limited influence on the broader anticoagulation strategy, whereas more specific prescribing patterns likely reflect a combination of patient characteristics and clinical considerations.

Patients with prior stroke/TIA represented a clinically higher-risk population, with a greater burden of cardiovascular comorbidities and impaired renal function. Similar profiles have been reported in contemporary AF registries, including RAFFINE, CARMEN-AF, COOL-AF, the Fushimi–Darlington registries, ETNA-AF-Europe, and GLORIA-AF [9,10,11,12,13,14]. These differences are important when interpreting prescribing patterns because anticoagulant choice in routine practice is rarely determined by a single characteristic. Age, renal function, heart failure, vascular disease, bleeding risk, concomitant treatment, and physician experience may all contribute. Differences between patients with and without prior cerebrovascular events therefore cannot be attributed to stroke/TIA history alone.

The evolution of anticoagulant prescribing observed in CRAFT is consistent with broader changes reported across different healthcare systems. Vora et al. described the rapid replacement of VKAs by DOACs across Europe and the United States, with an increasing role of apixaban [15]. Similar shifts toward factor Xa inhibitors, particularly apixaban, accompanied by declining dabigatran use have been reported in the Netherlands [16]. Changes in both DOAC uptake and dose selection have also been observed in nationwide Taiwanese data [17], while evolving anticoagulant prescribing has been documented in Polish and Swedish populations [18,19]. Real-world registries in specific clinical settings, such as the prospective IRIS registry of rivaroxaban use in patients undergoing AF ablation, further illustrate the evolution of anticoagulant use outside randomized clinical trials [20]. Our findings extend these observations into a more recent period, showing that changes in routine practice involve not only the replacement of VKAs by DOACs but also substantial shifts in prescribing within the DOAC class.

Whether prior stroke/TIA should influence these choices is less clear. Patients with AF and prior ischemic stroke/TIA are at particularly high risk of recurrent thromboembolic events, making effective anticoagulation a cornerstone of secondary prevention. However, current evidence provides limited support for selecting a particular DOAC or dose solely on the basis of prior stroke/TIA [21]. Current ESC guidelines similarly recommend DOACs for most eligible patients with AF requiring stroke prevention but do not favor a specific DOAC solely because of cerebrovascular history [8]. Against this background, the broadly parallel evolution of prescribing in patients with and without prior stroke/TIA is clinically plausible and suggests that changes in anticoagulant practice have been driven primarily by evolving evidence, guidelines, clinical experience, and treatment availability rather than by cerebrovascular history itself.

The differences observed at the level of individual DOACs and doses require more cautious interpretation. These associations may reflect how clinicians integrate cerebrovascular history with renal function, age, comorbidity burden, perceived bleeding risk, and familiarity with individual agents. The exploratory rivaroxaban dose finding warrants particular caution because the registry does not provide sufficient information to determine why a particular dose was prescribed or whether all drug-specific dose-reduction criteria were met. These findings should therefore be considered descriptive and hypothesis-generating rather than evidence favoring one DOAC or dose over another. The hierarchical approach used in the present analysis is particularly relevant in this context, as it evaluates successive levels of anticoagulant prescribing rather than treating individual regimens as unrelated outcomes.

From a clinical perspective, our findings support an individualized approach to anticoagulant prescribing rather than a uniform drug- or dose-selection strategy based on prior stroke/TIA alone. Differences among individual DOACs and doses should be viewed as features of real-world prescribing rather than evidence supporting a preferred therapeutic strategy. Treatment should continue to be individualized according to renal function, age, comorbidities, AF characteristics, and other relevant clinical factors. Importantly, this study evaluated prescribing patterns rather than clinical outcomes. Outcome-based studies are needed to determine whether the observed differences in prescribing are associated with recurrent thromboembolism, bleeding, or mortality.

### Limitations

Several limitations should be acknowledged. First, this was a retrospective, single-center observational study, which limits generalizability and leaves the possibility of residual confounding. Some potentially relevant variables, including atherosclerotic vascular disease and bleeding-related characteristics, were not consistently available throughout the study period and could not be included in the principal longitudinal models. Dose-specific analyses describe prescribing patterns rather than dose appropriateness because Cockcroft–Gault creatinine clearance and all drug-specific dose-reduction criteria could not be reliably reconstructed. Although FDR correction was applied to exploratory drug-specific comparisons, chance findings remain possible, particularly for associations that only narrowly retained statistical significance after correction.

The registry did not capture the clinical rationale for anticoagulant or dose selection, long-term adherence, treatment persistence, subsequent switching, or anticoagulant therapy at the time of later clinical events. Hospitalization was the unit of analysis, and approximately 20% of patients contributed more than one admission; patient-level cluster-robust standard errors were therefore used to account for within-patient correlation. Finally, clinical outcome data were not available, precluding assessment of comparative effectiveness or safety. Molecule- and dose-specific associations should therefore be considered hypothesis-generating and should not be interpreted as supporting or discouraging any particular anticoagulant regimen.

## 5. Conclusions

Anticoagulant prescribing changed substantially between 2017 and April 2026, with increasing OAC and DOAC use and marked shifts in prescribing within the DOAC class. Prior ischemic stroke/TIA was not independently associated with OAC use or DOAC versus VKA selection and did not significantly modify temporal prescribing trends, although differences were observed in individual DOAC selection and, in exploratory analyses, within-molecule dose selection. These findings suggest that prior stroke/TIA alone does not define a distinct anticoagulant prescribing pathway in contemporary clinical practice. Molecule- and dose-specific associations should be considered hypothesis-generating and require confirmation in outcome-based studies.

## Figures and Tables

**Table 1:** Baseline characteristics of hospitalizations according to prior ischemic stroke or transient ischemic attack.

Variable	No Prior Stroke/TIA (*n* = 3135)	Prior Stroke/TIA (*n* = 437)	*p* Value
**Demographics**			
Age, years	74.4 ± 11.2	76.2 ± 9.1	0.008
Male sex	1784/3135 (56.9%)	225/437 (51.5%)	0.037
**AF characteristics**			
CHA_2_DS_2_-VA	3.6 ± 1.4	6.1 ± 1.3	<0.001
LVEF, %	46.9 ± 12.8	46.3 ± 12.4	0.421
**Comorbidities**			
Hypertension	2198/3134 (70.1%)	331/437 (75.7%)	0.020
Diabetes mellitus	1049/3132 (33.5%)	202/437 (46.2%)	<0.001
Heart failure	2086/3118 (66.9%)	324/434 (74.7%)	0.002
Coronary artery disease	1078/3134 (34.4%)	185/437 (42.3%)	0.001
Previous myocardial infarction	590/3132 (18.8%)	112/436 (25.7%)	<0.001
PCI	598/3117 (19.2%)	84/431 (19.5%)	0.910
CABG	196/3129 (6.3%)	30/436 (6.9%)	0.670
COPD	314/3115 (10.1%)	48/434 (11.1%)	0.540
Atherosclerotic vascular disease	1189/2745 (43.3%)	201/390 (51.5%)	0.001
**Renal function**			
eGFR, mL/min	70.1 ± 21.4	63.8 ± 22.1	<0.001
**Concomitant medications**			
ASA	132/3127 (4.2%)	22/436 (5.0%)	0.504
Amiodarone	710/3135 (22.6%)	67/437 (15.3%)	<0.001
Statin	1815/3128 (58.0%)	311/437 (71.2%)	<0.001
ACE-I	1400/3127 (44.8%)	203/437 (46.5%)	0.541
ARB	619/3128 (19.8%)	89/437 (20.4%)	0.826
Spironolactone/Eplerenone	1355/3127 (43.3%)	210/437 (48.1%)	0.070
Diuretic	2374/3129 (75.9%)	350/437 (80.1%)	0.059
Beta-blocker	2651/3130 (84.7%)	373/436 (85.6%)	0.693
SGLT-2i	568/1227 (46.3%)	95/168 (56.5%)	0.016
Entresto	143/1230 (11.6%)	22/169 (13.0%)	0.690

Data are presented as mean ± SD or n/N (%). Continuous variables were compared using the Mann–Whitney U test and categorical variables using the χ^2^ test. Missing values were treated as unavailable and excluded from the respective analyses; percentages were calculated using observations with available data as the denominator. Abbreviations: ACE-I, angiotensin-converting enzyme inhibitor; AF, atrial fibrillation; ARB, angiotensin receptor blocker; ASA, acetylsalicylic acid; CABG, coronary artery bypass grafting; COPD, chronic obstructive pulmonary disease; eGFR, estimated glomerular filtration rate; LVEF, left ventricular ejection fraction; PCI, percutaneous coronary intervention; SD, standard deviation; SGLT-2i, sodium-glucose cotransporter-2 inhibitor; TIA, transient ischemic attack.

**Table 2:** Unadjusted anticoagulant prescribing according to prior stroke/TIA status.

Treatment	No Prior Stroke/TIA	Prior Stroke/TIA	*p* Value
VKA	7.4%	11.4%	0.004
DOAC (overall)	85.0%	81.4%	0.063
Dabigatran 150 mg	9.2%	11.9%	0.088
Dabigatran 110 mg	6.8%	10.5%	0.007
Rivaroxaban 20 mg	22.2%	12.4%	<0.001
Rivaroxaban 15 mg	13.1%	15.1%	0.283
Apixaban 5 mg	15.9%	13.3%	0.180
Apixaban 2.5 mg	17.6%	18.3%	0.759

Data are presented as percentages. Comparisons were performed using the χ^2^ test. Percentages were calculated using all hospitalizations within each prior stroke/TIA group as the denominator. VKA and overall DOAC use do not sum to 100% because some hospitalizations resulted in discharge without oral anticoagulation. Abbreviations: DOAC, direct oral anticoagulant; TIA, transient ischemic attack; VKA, vitamin K antagonist.

**Table 3:** Hierarchical analysis of the association between prior ischemic stroke/TIA and anticoagulant prescribing.

Prescribing Level	Comparison	Adjusted Effect Estimate	95% CI	*p* Value	FDR-Adjusted *q* Value
OAC use	OAC vs. no OAC	OR 1.099	0.738–1.637	0.643	—
OAC class	DOAC vs. VKA among OAC	OR 0.676	0.453–1.009	0.055	—
DOAC molecule	Global molecule selection	Wald χ^2^ = 13.22, df = 2	—	0.0013	—
DOAC molecule	Dabigatran vs. apixaban	RRR 1.870	1.286–2.718	0.0010	0.0052
DOAC molecule	Rivaroxaban vs. apixaban	RRR 1.025	0.740–1.419	0.882	0.927
Within-molecule dose	Dabigatran 150 vs. 110 mg	OR 1.304	0.738–2.306	0.361	0.602
Within-molecule dose	Rivaroxaban 20 vs. 15 mg	OR 0.542	0.326–0.904	0.0189	0.0471
Within-molecule dose	Apixaban 5 vs. 2.5 mg	OR 1.023	0.624–1.678	0.927	0.927

Models assessed prior stroke/TIA as the exposure of interest and included calendar year, age, sex, hypertension, diabetes mellitus, heart failure, AF type, and eGFR as covariates, with patient-level cluster-robust standard errors. Analysis-specific sample sizes were 3526 for OAC versus no OAC, 3242 for DOAC versus VKA, 2979 for DOAC molecule selection, 588 for dabigatran dose, 1203 for rivaroxaban dose, and 1174 for apixaban dose. Apixaban was the reference category for DOAC molecule selection; rivaroxaban 10 mg was excluded from the 20-versus-15-mg comparison. Benjamini–Hochberg FDR correction was applied to the five exploratory drug-specific comparisons (two molecule and three within-molecule dose contrasts). Dose-specific analyses describe prescribing patterns rather than dose appropriateness. Abbreviations: CI, confidence interval; DOAC, direct oral anticoagulant; eGFR, estimated glomerular filtration rate; FDR, false discovery rate; OAC, oral anticoagulant; OR, odds ratio; RRR, relative risk ratio; TIA, transient ischemic attack; VKA, vitamin K antagonist.

**Table 4:** Temporal trends in hierarchical anticoagulant prescribing.

Prescribing Level	Comparison	Effect per Calendar Year	95% CI	*p* Value	FDR-Adjusted *q* Value
OAC use	OAC vs. no OAC	OR/year 1.160	1.095–1.229	<0.001	—
OAC class	DOAC vs. VKA among OAC	OR/year 1.303	1.232–1.378	<0.001	—
DOAC molecule	Global molecule selection	Wald χ^2^ = 131.61, df = 2	—	<0.001	—
DOAC molecule	Dabigatran vs. apixaban	RRR/year 0.772	0.737–0.808	<0.001	<0.001
DOAC molecule	Rivaroxaban vs. apixaban	RRR/year 0.865	0.833–0.898	<0.001	<0.001
Within-molecule dose	Dabigatran 150 vs. 110 mg	OR/year 1.062	0.974–1.159	0.174	0.174
Within-molecule dose	Rivaroxaban 20 vs. 15 mg	OR/year 1.147	1.077–1.221	<0.001	<0.001
Within-molecule dose	Apixaban 5 vs. 2.5 mg	OR/year 1.396	1.298–1.502	<0.001	<0.001

Models were adjusted for age, sex, hypertension, diabetes mellitus, heart failure, AF type, eGFR, and prior stroke/TIA, with patient-level cluster-robust standard errors. Calendar year was modeled as a continuous variable. Analysis-specific denominators followed the same hierarchical definitions as in Table 3. Benjamini–Hochberg FDR correction was applied to the five exploratory drug-specific comparisons. Dose-specific analyses describe prescribing patterns rather than dose appropriateness. Abbreviations: CI, confidence interval; DOAC, direct oral anticoagulant; eGFR, estimated glomerular filtration rate; FDR, false discovery rate; OAC, oral anticoagulant; OR, odds ratio; RRR, relative risk ratio; TIA, transient ischemic attack; VKA, vitamin K antagonist.

## Data Availability

The datasets used and analyzed during the current study are available from the corresponding author upon reasonable request.

## Supplementary Materials

The following supporting information can be downloaded at: https://www.mdpi.com/article/10.3390/jcm15187242/s1, Table S1: Interaction Between Calendar Year and Prior Stroke/TIA Across Hierarchical Anticoagulant Prescribing; Table S2: Sensitivity Analyses of Temporal Trends in Anticoagulant Prescribing; Table S3: Annual Anticoagulant Prescribing Patterns by Prior Ischemic Stroke/TIA Status; Table S4: Complete-case sensitivity analysis including atherosclerotic vascular disease.

## Abbreviations

ACE-I	angiotensin-converting enzyme inhibitor
AF	atrial fibrillation
ARB	angiotensin receptor blocker
ASA	acetylsalicylic acid
CABG	coronary artery bypass grafting
CHA_2_DS_2_-VA	congestive heart failure, hypertension, age ≥75 years (2 points), diabetes mellitus, prior stroke/transient ischemic attack/thromboembolism (2 points), vascular disease, and age 65–74 years
CI	confidence interval
COPD	chronic obstructive pulmonary disease
CRAFT	MultiCenter expeRience in AFib patients Treated with OAC
DOAC	direct oral anticoagulant
eGFR	estimated glomerular filtration rate
FDR	false discovery rate
LVEF	left ventricular ejection fraction
OAC	oral anticoagulant
OR	odds ratio
PCI	percutaneous coronary intervention
RRR	relative risk ratio
SD	standard deviation
SGLT-2i	sodium-glucose cotransporter-2 inhibitor
TIA	transient ischemic attack
VKA	vitamin K antagonist
