# Supplementary material for: Temporal Trends in Anticoagulant Prescribing in Patients with Atrial Fibrillation According to Prior Ischemic Stroke or Transient Ischemic Attack: Insights from the CRAFT Registry
Source: J Clin Med. 2026 Sep 17;15(18):7242. doi: 10.3390/jcm15187242 (PMC13607917; doi:10.3390/jcm15187242)
Supplement: Supplementary file 1 [file jcm-15-07242-s001.zip › jcm-4529988-supplementary.pdf]

Supplementary Table S1. Interaction Between Calendar Year and Prior Stroke/TIA Across Hierarchical Anticoagulant Prescribing

Prescribing level	Comparison	Adjusted interaction estimate	95% CI	*p* value	FDR-adjusted *q* value
OAC use	OAC vs. no OAC	OR 0.952	0.816–1.111	0.535	—
OAC class	DOAC vs. VKA	OR 0.904	0.785–1.042	0.165	—
DOAC molecule	Overall molecule selection	Wald $\chi^2 = 3.25$	df = 2	0.197	—
DOAC molecule	Dabigatran vs. apixaban	RRR 1.110	0.976–1.261	0.112	0.297
DOAC molecule	Rivaroxaban vs. apixaban	RRR 1.088	0.970–1.220	0.152	0.297
Within-molecule dose	Dabigatran 150 vs. 110 mg	OR 1.127	0.924–1.375	0.237	0.297
Within-molecule dose	Rivaroxaban 20 vs. 15 mg	OR 1.046	0.874–1.251	0.625	0.625
Within-molecule dose	Apixaban 5 vs. 2.5 mg	OR 0.887	0.730–1.077	0.227	0.297

Interaction estimates correspond to the calendar year × prior ischemic stroke/TIA term and assess whether temporal prescribing trends differed according to prior stroke/TIA status. Models were adjusted for age, sex, hypertension, diabetes mellitus, heart failure, AF type, and eGFR and included the main effects of calendar year and prior ischemic stroke/TIA. Patient-level cluster-robust standard errors were used to account for repeated hospitalizations. Apixaban was the reference category in the multinomial DOAC molecule model. Benjamini–Hochberg FDR correction was applied to the five exploratory molecule- and dose-specific interaction contrasts. Dose-specific analyses describe prescribing patterns rather than dose appropriateness. Rivaroxaban 10 mg was excluded from the 20-versus-15-mg comparison. No significant interaction was observed. All *p* values and FDR-adjusted *q* values are two-sided.

Abbreviations: AF, atrial fibrillation; CI, confidence interval; DOAC, direct oral anticoagulant; eGFR, estimated glomerular filtration rate; FDR, false discovery rate; OAC, oral anticoagulant; OR, odds ratio; RRR, relative risk ratio; TIA, transient ischemic attack; VKA, vitamin K antagonist.

Supplementary Table S2. Sensitivity Analyses of Temporal Trends in Anticoagulant Prescribing

Prescribing comparison	Primary continuous-year model	Calendar year categorical: global test	Continuous-year model excluding Jan–Apr 2026
OAC vs no OAC	OR 1.160 (1.095–1.229); $p < 0.001$	$\chi^2 = 43.54$, df = 8; $p < 0.001$	OR/year 1.179 (1.110–1.252); $p < 0.001$
DOAC vs VKA	OR 1.303 (1.232–1.378); $p < 0.001$	$\chi^2 = 88.09$, df = 8; $p < 0.001$	OR/year 1.294 (1.221–1.372); $p < 0.001$
Dabigatran vs apixaban	RRR 0.772 (0.737–0.808); $p < 0.001$	$\chi^2 = 164.24$, df = 16; $p < 0.001$	RRR/year 0.771 (0.734–0.809); $p < 0.001$
Rivaroxaban vs apixaban	RRR 0.865 (0.833–0.898); $p < 0.001$	$\chi^2 = 164.24$, df = 16; $p < 0.001$	RRR/year 0.850 (0.817–0.885); $p < 0.001$
Dabigatran 150 vs 110	OR 1.062 (0.974–1.159); $p = 0.174$	$\chi^2 = 21.50$, df = 8; $p = 0.006$	OR/year 1.044 (0.952–1.145); $p = 0.364$
Rivaroxaban 20 vs 15	OR 1.147 (1.077–1.221); $p < 0.001$	$\chi^2 = 24.38$, df = 8; $p = 0.002$	OR/year 1.143 (1.068–1.222); $p < 0.001$
Apixaban 5 vs 2.5	OR 1.396 (1.298–1.502); $p < 0.001$	$\chi^2 = 82.98$, df = 8; $p < 0.001$	OR/year 1.389 (1.285–1.501); $p < 0.001$

Primary continuous-year estimates represent the change per one-calendar-year increase. The categorical-year sensitivity analysis treated calendar year as a factor (2017 as the reference) and therefore did not assume a linear temporal trajectory; the table reports the global Wald test for calendar year. No value was imputed for 2023 because registry recruitment was suspended. The second sensitivity analysis repeated the continuous-year models after excluding all January–April 2026 observations. All adjusted models used patient-level cluster-robust standard errors to account for repeated hospitalizations. For DOAC molecule selection, the primary continuous-year global temporal effect was significant (Wald $\chi^2 = 131.61$, df = 2, $p < 0.001$); the categorical-year global test was also significant (Wald $\chi^2 = 164.24$, df = 16, $p < 0.001$); and after exclusion of 2026, the global temporal effect remained significant (Wald $\chi^2 = 122.73$, df = 2, $p < 0.001$). Dose-specific analyses describe observed prescribing patterns rather than dose appropriateness. Rivaroxaban 10 mg was excluded from the 20-versus-15-mg comparison.

Abbreviations: CI, confidence interval; DOAC, direct oral anticoagulant; OAC, oral anticoagulant; OR, odds ratio; RRR, relative risk ratio; VKA, vitamin K antagonist.

Supplementary Table S3. Annual Anticoagulant Prescribing Patterns by Prior Ischemic Stroke/TIA Status

OAC use

Year	No prior stroke/TIA, n/N (%; 95% CI)	Prior stroke/TIA, n/N (%; 95% CI)
2017	237/280 (84.6%; 80.0–88.4)	35/41 (85.4%; 71.6–93.1)
2018	272/313 (86.9%; 82.7–90.2)	35/40 (87.5%; 73.9–94.5)
2019	219/247 (88.7%; 84.1–92.0)	44/47 (93.6%; 82.8–97.8)
2020	229/258 (88.8%; 84.3–92.1)	30/35 (85.7%; 70.6–93.7)
2021	295/328 (89.9%; 86.2–92.7)	34/36 (94.4%; 81.9–98.5)
2022	452/475 (95.2%; 92.8–96.8)	67/69 (97.1%; 90.0–99.2)
2024	628/655 (95.9%; 94.1–97.2)	82/86 (95.3%; 88.6–98.2)
2025	413/433 (95.4%; 93.0–97.0)	58/63 (92.1%; 82.7–96.6)
2026 (Jan–Apr)	141/150 (94.0%; 89.0–96.8)	19/20 (95.0%; 76.4–99.1)

DOAC among classifiable OAC-treated hospitalizations

Year	No prior stroke/TIA, n/N (%; 95% CI)	Prior stroke/TIA, n/N (%; 95% CI)
2017	193/237 (81.4%; 76.0–85.9)	23/35 (65.7%; 49.2–79.2)
2018	225/272 (82.7%; 77.8–86.7)	30/35 (85.7%; 70.6–93.7)
2019	197/219 (90.0%; 85.3–93.3)	40/44 (90.9%; 78.8–96.4)
2020	210/229 (91.7%; 87.4–94.6)	29/30 (96.7%; 83.3–99.4)
2021	265/295 (89.8%; 85.9–92.8)	27/34 (79.4%; 63.2–89.7)
2022	413/452 (91.4%; 88.4–93.6)	56/67 (83.6%; 72.9–90.6)
2024	620/628 (98.7%; 97.5–99.4)	78/82 (95.1%; 88.1–98.1)
2025	404/413 (97.8%; 95.9–98.8)	55/58 (94.8%; 85.9–98.2)
2026 (Jan–Apr)	141/141 (100.0%; 97.3–100.0)	18/19 (94.7%; 75.4–99.1)

Dabigatran among classifiable DOAC-treated hospitalizations

Year	No prior stroke/TIA, n/N (%; 95% CI)	Prior stroke/TIA, n/N (%; 95% CI)
2017	62/193 (32.1%; 25.9–39.0)	10/23 (43.5%; 25.6–63.2)
2018	51/225 (22.7%; 17.7–28.6)	9/30 (30.0%; 16.7–47.9)
2019	52/197 (26.4%; 20.7–33.0)	18/40 (45.0%; 30.7–60.2)
2020	42/210 (20.0%; 15.2–25.9)	6/29 (20.7%; 9.8–38.4)
2021	61/265 (23.0%; 18.4–28.5)	5/27 (18.5%; 8.2–36.7)

Year	No prior stroke/TIA, n/N (%; 95% CI)	Prior stroke/TIA, n/N (%; 95% CI)
2022	73/413 (17.7%; 14.3–21.6)	11/56 (19.6%; 11.3–31.8)
2024	108/620 (17.4%; 14.6–20.6)	25/78 (32.1%; 22.7–43.0)
2025	44/404 (10.9%; 8.2–14.3)	9/55 (16.4%; 8.9–28.3)
2026 (Jan–Apr)	9/141 (6.4%; 3.4–11.7)	4/18 (22.2%; 9.0–45.2)

Rivaroxaban among classifiable DOAC-treated hospitalizations

Year	No prior stroke/TIA, n/N (%; 95% CI)	Prior stroke/TIA, n/N (%; 95% CI)
2017	92/193 (47.7%; 40.7–54.7)	5/23 (21.7%; 9.7–41.9)
2018	118/225 (52.4%; 45.9–58.9)	15/30 (50.0%; 33.2–66.8)
2019	84/197 (42.6%; 35.9–49.6)	13/40 (32.5%; 20.1–48.0)
2020	94/210 (44.8%; 38.2–51.5)	8/29 (27.6%; 14.7–45.7)
2021	103/265 (38.9%; 33.2–44.9)	8/27 (29.6%; 15.9–48.5)
2022	137/413 (33.2%; 28.8–37.8)	21/56 (37.5%; 26.0–50.6)
2024	228/620 (36.8%; 33.1–40.6)	17/78 (21.8%; 14.1–32.2)
2025	188/404 (46.5%; 41.7–51.4)	27/55 (49.1%; 36.4–61.9)
2026 (Jan–Apr)	73/141 (51.8%; 43.6–59.9)	7/18 (38.9%; 20.3–61.4)

Apixaban among classifiable DOAC-treated hospitalizations

Year	No prior stroke/TIA, n/N (%; 95% CI)	Prior stroke/TIA, n/N (%; 95% CI)
2017	39/193 (20.2%; 15.1–26.4)	8/23 (34.8%; 18.8–55.1)
2018	56/225 (24.9%; 19.7–30.9)	6/30 (20.0%; 9.5–37.3)
2019	61/197 (31.0%; 24.9–37.7)	9/40 (22.5%; 12.3–37.5)
2020	74/210 (35.2%; 29.1–41.9)	15/29 (51.7%; 34.4–68.6)
2021	101/265 (38.1%; 32.5–44.1)	14/27 (51.9%; 34.0–69.3)
2022	203/413 (49.2%; 44.4–54.0)	24/56 (42.9%; 30.8–55.9)
2024	284/620 (45.8%; 41.9–49.7)	36/78 (46.2%; 35.5–57.1)
2025	172/404 (42.6%; 37.8–47.4)	19/55 (34.5%; 23.4–47.7)
2026 (Jan–Apr)	59/141 (41.8%; 34.0–50.1)	7/18 (38.9%; 20.3–61.4)

Dabigatran 150 mg among 150/110 mg prescriptions

Year	No prior stroke/TIA, n/N (%; 95% CI)	Prior stroke/TIA, n/N (%; 95% CI)
2017	28/62 (45.2%; 33.4–57.5)	1/10 (10.0%; 1.8–40.4)

Year	No prior stroke/TIA, n/N (%; 95% CI)	Prior stroke/TIA, n/N (%; 95% CI)
2018	33/51 (64.7%; 51.0–76.4)	2/9 (22.2%; 6.3–54.7)
2019	33/52 (63.5%; 49.9–75.2)	11/18 (61.1%; 38.6–79.7)
2020	24/42 (57.1%; 42.2–70.9)	5/6 (83.3%; 43.6–97.0)
2021	31/61 (50.8%; 38.6–62.9)	3/5 (60.0%; 23.1–88.2)
2022	35/73 (47.9%; 36.9–59.2)	5/11 (45.5%; 21.3–72.0)
2024	65/108 (60.2%; 50.8–68.9)	14/25 (56.0%; 37.1–73.3)
2025	32/44 (72.7%; 58.2–83.7)	6/9 (66.7%; 35.4–87.9)
2026 (Jan–Apr)	7/9 (77.8%; 45.3–93.7)	4/4 (100.0%; 51.0–100.0)

Rivaroxaban 20 mg among 20/15 mg prescriptions

Year	No prior stroke/TIA, n/N (%; 95% CI)	Prior stroke/TIA, n/N (%; 95% CI)
2017	41/89 (46.1%; 36.1–56.4)	1/4 (25.0%; 4.6–69.9)
2018	57/117 (48.7%; 39.8–57.7)	3/15 (20.0%; 7.0–45.2)
2019	38/81 (46.9%; 36.4–57.7)	3/13 (23.1%; 8.2–50.3)
2020	43/93 (46.2%; 36.5–56.3)	4/8 (50.0%; 21.5–78.5)
2021	54/100 (54.0%; 44.3–63.4)	5/8 (62.5%; 30.6–86.3)
2022	87/137 (63.5%; 55.2–71.1)	7/21 (33.3%; 17.2–54.6)
2024	166/226 (73.5%; 67.3–78.8)	13/17 (76.5%; 52.7–90.4)
2025	146/188 (77.7%; 71.2–83.0)	14/27 (51.9%; 34.0–69.3)
2026 (Jan–Apr)	62/73 (84.9%; 75.0–91.4)	4/7 (57.1%; 25.0–84.2)

Apixaban 5 mg among 5/2.5 mg prescriptions

Year	No prior stroke/TIA, n/N (%; 95% CI)	Prior stroke/TIA, n/N (%; 95% CI)
2017	2/39 (5.1%; 1.4–16.9)	3/8 (37.5%; 13.7–69.4)
2018	4/56 (7.1%; 2.8–17.0)	0/6 (0.0%; 0.0–39.0)
2019	16/61 (26.2%; 16.8–38.4)	3/9 (33.3%; 12.1–64.6)
2020	15/74 (20.3%; 12.7–30.8)	5/15 (33.3%; 15.2–58.3)
2021	42/101 (41.6%; 32.5–51.3)	3/14 (21.4%; 7.6–47.6)
2022	87/203 (42.9%; 36.2–49.7)	9/24 (37.5%; 21.2–57.3)
2024	165/284 (58.1%; 52.3–63.7)	20/36 (55.6%; 39.6–70.5)
2025	127/172 (73.8%; 66.8–79.8)	8/19 (42.1%; 23.1–63.7)

Year	No prior stroke/TIA, n/N (%; 95% CI)	Prior stroke/TIA, n/N (%; 95% CI)
2026 (Jan–Apr)	42/59 (71.2%; 58.6–81.2)	7/7 (100.0%; 64.6–100.0)

Observed annual proportions are presented as n/N (%; 95% CI), with 95% confidence intervals calculated using the Wilson score method. No observations are shown for 2023 because registry recruitment was suspended. Data for 2026 include January through April only. Denominators are analysis-specific and therefore differ across prescribing levels: OAC use was assessed among hospitalizations with classifiable OAC status; DOAC versus VKA among classifiable OAC-treated hospitalizations classifiable as receiving one of these OAC classes; molecule proportions among DOAC-treated hospitalizations with a single classifiable DOAC molecule; and dose proportions within the corresponding classifiable dose pair. Rivaroxaban 10 mg was excluded from the 20-versus-15-mg dose comparison. These annual estimates are descriptive and complement the adjusted temporal models.

Abbreviations: CI, confidence interval; DOAC, direct oral anticoagulant; OAC, oral anticoagulant; TIA, transient ischemic attack; VKA, vitamin K antagonist.

Supplementary Table S4. Complete-Case Sensitivity Analysis Including Atherosclerotic Vascular Disease

Analysis	Prescribing comparison	N	Adjusted effect estimate	95% CI	*p* value	FDR-adjusted *q* value
**A. Association with prior stroke/TIA**						
	OAC vs. no OAC	3094	OR 1.140	0.759–1.712	0.527	—
	DOAC vs. VKA	2822	OR 0.673	0.446–1.014	0.058	—
	Overall DOAC molecule selection	2569	Wald χ^2 = 11.06; df = 2	—	0.0040	—
	Dabigatran vs. apixaban	2569	RRR 1.785	1.208–2.639	0.0036	0.0182
	Rivaroxaban vs. apixaban	2569	RRR 1.005	0.713–1.417	0.975	0.975
	Dabigatran 150 mg vs. 110 mg	542	OR 1.266	0.681–2.355	0.456	0.760
	Rivaroxaban 20 mg vs. 15 mg	985	OR 0.525	0.307–0.896	0.0182	0.0455
	Apixaban 5 mg vs. 2.5 mg	1028	OR 0.913	0.543–1.536	0.733	0.916
**B. Temporal trend**						
	OAC vs. no OAC	3094	OR/year 1.162	1.092–1.237	< 0.001	—

Analysis	Prescribing comparison	N	Adjusted effect estimate	95% CI	*p* value	FDR-adjusted *q* value
	DOAC vs. VKA	2822	OR/year 1.296	1.219–1.379	< 0.001	—
	Overall DOAC molecule selection	2569	Wald χ^2 = 109.07; df = 2	—	< 0.001	—
	Dabigatran vs. apixaban	2569	RRR/year 0.777	0.738–0.819	< 0.001	< 0.001
	Rivaroxaban vs. apixaban	2569	RRR/year 0.840	0.805–0.877	< 0.001	< 0.001
	Dabigatran 150 mg vs. 110 mg	542	OR/year 1.048	0.952–1.154	0.336	0.336
	Rivaroxaban 20 mg vs. 15 mg	985	OR/year 1.155	1.074–1.242	< 0.001	< 0.001
	Apixaban 5 mg vs. 2.5 mg	1028	OR/year 1.407	1.296–1.527	< 0.001	< 0.001
**C. Calendar year × prior stroke/TIA interaction**						
	OAC vs. no OAC	3094	OR 0.969	0.819–1.146	0.716	—
	DOAC vs. VKA	2822	OR 0.928	0.796–1.083	0.344	—
	Overall DOAC molecule selection	2569	Wald χ^2 = 3.02; df = 2	—	0.221	—
	Dabigatran vs. apixaban	2569	RRR 1.084	0.940–1.249	0.268	0.343
	Rivaroxaban vs. apixaban	2569	RRR 1.113	0.982–1.262	0.0935	0.343
	Dabigatran 150 mg vs. 110 mg	542	OR 1.134	0.905–1.421	0.274	0.343
	Rivaroxaban 20 mg vs. 15 mg	985	OR 1.063	0.877–1.288	0.536	0.536
	Apixaban 5 mg vs. 2.5 mg	1028	OR 0.871	0.711–1.067	0.183	0.343

Complete-case sensitivity analyses additionally included atherosclerotic vascular disease (AVD) in the covariate set. Models were otherwise specified identically to the principal analyses and adjusted for calendar year, prior ischemic stroke/TIA, age, sex, hypertension, diabetes mellitus, heart failure, AF type, and eGFR, as applicable. Patient-level cluster-robust standard errors were used to account for repeated hospitalizations. For temporal-trend models, effect estimates represent change per one-calendar-year increase; interaction models included the

corresponding main effects of calendar year and prior stroke/TIA. Apixaban was the reference category in multinomial DOAC molecule models. Benjamini–Hochberg FDR correction was applied separately to the five exploratory molecule- and dose-specific comparisons in each analysis. Rivaroxaban 10 mg was excluded from the 20-versus-15-mg comparison. Dose-specific analyses describe observed prescribing patterns rather than dose appropriateness.

Abbreviations: AF, atrial fibrillation; AVD, atherosclerotic vascular disease; CI, confidence interval; DOAC, direct oral anticoagulant; eGFR, estimated glomerular filtration rate; FDR, false discovery rate; OAC, oral anticoagulant; OR, odds ratio; RRR, relative risk ratio; TIA, transient ischemic attack; VKA, vitamin K antagonist.
